# A Manual of Dietetics

**Published:** 1886-11

**Authors:** 


A MANUAL OF DIETETICS. By J. Milner Fothergill, M. D., Edin., Physician to the City of London Hospital for Diseases of the Chest (Victoria Park); Hon. M. D. Rush

Medical College, Chicago, Ill.; Foreign Associate Fellow of the College of Physicians, Philadelphia. 8vo, extra muslin, 255 pages. Price $2.50. New York: William Wood and Company.

This book treats of a subject which has been and is now too much neglected by the teachers in our medical colleges. It is as important that proper food should be given as that proper medicines should be administered, and the sooner this fact is recognized the better will it be for our sick. The author very properly condemns the much vaunted beef tea as a food, in the following pointed language: "The mistaken views about the nutritive value of beef tea have been murderous. All the blood-shed caused by the war-like ambition of Napoleon is as nothing compared to myriads of persons who have sunk into their graves from a misplaced confidence in the food value of beef tea. As a food, it is but as the mirage of water seen by the thirsty traveler in the desert; there is no real water. So with beef tea—it is not a food." Many false ideas of feeding the sick are exposed in the work. It is a valuable work, and should be in the hands of every physician in the land.

